# RETRACTION: Wound Infection Prevention Strategies in Colorectal Endoscopic Mucosal Resection: A Meta‐Analysis of Prophylactic Measures

**DOI:** 10.1111/iwj.70480

**Published:** 2025-04-02

## Abstract

**RETRACTION:** QiH. , WangZ. , ShenF. , YuW. , DuanS. , LiX. , and HuangX. , “Wound Infection Prevention Strategies in Colorectal Endoscopic Mucosal Resection: A Meta‐Analysis of Prophylactic Measures,” International Wound Journal 21, no. 1 (2024): e14544, 10.1111/iwj.14544.38272812 PMC10789545

The above article, published online on 15 January 2024, in Wiley Online Library (http://onlinelibrary.wiley.com/), has been retracted by agreement between the journal Editor in Chief, Professor Keith Harding; and John Wiley & Sons Ltd. Following an investigation by the publisher, all parties have concluded that this article was accepted solely on the basis of a compromised peer review process. The editors have therefore decided to retract the article. The authors did not respond to our notice regarding the retraction.

**RETRACTION:** H. Qi , Z. Wang , F. Shen , W. Yu , S. Duan , X. Li , and X. Huang , “Wound Infection Prevention Strategies in Colorectal Endoscopic Mucosal Resection: A Meta‐Analysis of Prophylactic Measures,” International Wound Journal 21, no. 1 (2024): e14544, 10.1111/iwj.14544.38272812 PMC10789545

The above article, published online on 15 January 2024, in Wiley Online Library (http://onlinelibrary.wiley.com/), has been retracted by agreement between the journal Editor in Chief, Professor Keith Harding; and John Wiley & Sons Ltd. Following an investigation by the publisher, all parties have concluded that this article was accepted solely on the basis of a compromised peer review process. The editors have therefore decided to retract the article. The authors did not respond to our notice regarding the retraction.

